# Artificial Intelligence–Assisted System in Postoperative Follow-up of Orthopedic Patients: Exploratory Quantitative and Qualitative Study

**DOI:** 10.2196/16896

**Published:** 2020-05-26

**Authors:** Yanyan Bian, Yongbo Xiang, Bingdu Tong, Bin Feng, Xisheng Weng

**Affiliations:** 1 Department of Orthopedic Surgery Peking Union Medical College Hospital Chinese Academy of Medical Science and Peking Union Medical College Beijing China

**Keywords:** artificial intelligence, conversational agent, follow-up, cost-effectiveness

## Abstract

**Background:**

Patient follow-up is an essential part of hospital ward management. With the development of deep learning algorithms, individual follow-up assignments might be completed by artificial intelligence (AI). We developed an AI-assisted follow-up conversational agent that can simulate the human voice and select an appropriate follow-up time for quantitative, automatic, and personalized patient follow-up. Patient feedback and voice information could be collected and converted into text data automatically.

**Objective:**

The primary objective of this study was to compare the cost-effectiveness of AI-assisted follow-up to manual follow-up of patients after surgery. The secondary objective was to compare the feedback from AI-assisted follow-up to feedback from manual follow-up.

**Methods:**

The AI-assisted follow-up system was adopted in the Orthopedic Department of Peking Union Medical College Hospital in April 2019. A total of 270 patients were followed up through this system. Prior to that, 2656 patients were followed up by phone calls manually. Patient characteristics, telephone connection rate, follow-up rate, feedback collection rate, time spent, and feedback composition were compared between the two groups of patients.

**Results:**

There was no statistically significant difference in age, gender, or disease between the two groups. There was no significant difference in telephone connection rate (manual: 2478/2656, 93.3%; AI-assisted: 249/270, 92.2%; *P*=.50) or successful follow-up rate (manual: 2301/2478, 92.9%; AI-assisted: 231/249, 92.8%; *P*=.96) between the two groups. The time spent on 100 patients in the manual follow-up group was about 9.3 hours. In contrast, the time spent on the AI-assisted follow-up was close to 0 hours. The feedback rate in the AI-assisted follow-up group was higher than that in the manual follow-up group (manual: 68/2656, 2.5%; AI-assisted: 28/270, 10.3%; *P*<.001). The composition of feedback was different in the two groups. Feedback from the AI-assisted follow-up group mainly included nursing, health education, and hospital environment content, while feedback from the manual follow-up group mostly included medical consultation content.

**Conclusions:**

The effectiveness of AI-assisted follow-up was not inferior to that of manual follow-up. Human resource costs are saved by AI. AI can help obtain comprehensive feedback from patients, although its depth and pertinence of communication need to be improved.

## Introduction

Artificial intelligence (AI) is a system that can correctly interpret external data, learn from such data, and flexibly apply the acquired knowledge to achieve specific tasks and goals. With the dramatic improvement of computer power to process big data, AI is already ubiquitous in our daily life in the past 50 years. In the past several years, the application prospect of AI in surgery, radiology, dermatology, and oncology is up-and-coming [1-3].

Telemedicine is a medical method used to provide clinical and educational services for remote areas. Information and communication technology are used to transmit medical information [4,5]. Telemedicine attempts to overcome challenges in health services delivery due to distance, time, and rough terrain by improving cost-effectiveness and accessibility of health services in both developing and developed regions [6]. It is an “open and evolving science that incorporates advancements in new technologies and adapts to the changing health demands and social environments.” [7] With the growth of AI and big data analytics, the scope and capability of telemedicine have expanded in recent years. Current telemedicine applications can be divided into four categories: patient monitoring, medical information technology, AI-assisted diagnosis, and information analysis collaboration [7]. With the assistance of AI, telemedicine might be an effective method for disease assessment, diagnosis, management, and monitoring [6], especially in chronic disease [8-12], skin diseases [13], and postoperative follow-up care [14].

Postoperative follow-up is an essential part of orthopedic surgery. Medical institutions can provide service for discharged postoperative patients through follow-up. Traditional methods include phone calls, emails, visiting, and reexamination at clinic; all of these methods need a lot of medical resources. With the development of AI and telemedicine, computers or mobile phones could be used to complete more and more of the follow-up work.

In this paper, we describe an AI-assisted system for the follow-up of patients after surgery. The study’s aim was to compare the cost-effectiveness and feedback composition of the AI-assisted system with a traditional method for postoperative follow-up.

## Methods

### Ethics Approval and Consent to Participate

This study was approved by the Research and Ethics Institutional Committee of Peking Union Medical College Hospital (PUMCH).

### Patient Enrollment and Data Collection

The AI-assisted follow-up system was launched in the Orthopedic Department of PUMCH in 2019. We enrolled 270 patients who had undergone orthopedic surgery at PUMCH from April 2019 to May 2019. This group of patients was defined as the AI-assisted follow-up group since all of the postoperative follow-up was completed by an AI-assisted follow-up system. 2,656 patients who had undergone orthopedic surgery at PUMCH from April 2018 to March 2019 were enrolled as the manual follow-up group. The postoperative follow-up of this group was completed by manual phone calls. Patient characteristics for both groups were collected from the hospital information management system including gender, age, and disease type.

### AI-Assisted Follow-Up System

**Figure 1:**
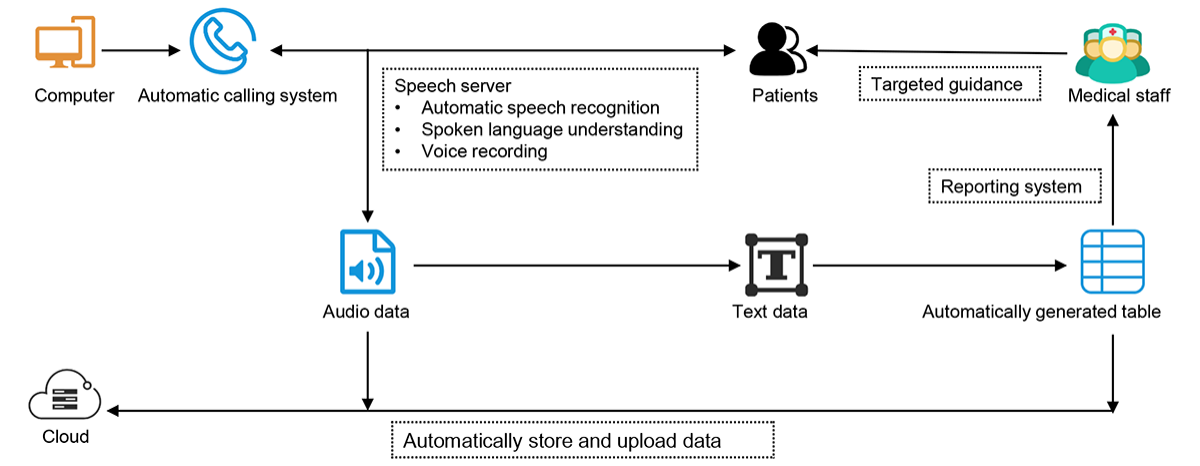
Block diagram of the AI-assisted follow-up system.

Figure 1 presents a block diagram of the AI-assisted follow-up system. The AI-assisted follow-up system obtained baseline information for each discharged patient including the ID number, gender, age, discharge date, diagnosis, telephone number, and caller location. The system called patients via automated speech telephony delivery in batches from 8:30 AM to 8:30 PM every day allowing hundreds of calls to be made daily. Interactions between the system and patients were based on machine learning, speech recognition, spoken language understanding, and human voice simulation technology. Communication contents included patient satisfaction in the hospital environment, nursing, and health education; wound recovery; functional training; postoperative complications; and other surgery-related medical consulting. The system was able to identify dialects in different parts of China via speech recognition technology and voice information was converted into text in real time. An example of transcribed dialogue that was automatically converted into text is shown in Textbox 1. A report, shown in Table 1, was generated and automatically uploaded to the cloud afterward. Surgeons and nurses could review the report and respond to patient feedback, if necessary.

**Transcription of a sample dialogue.**

System: Hello, this is Peking union medical college hospital. Could you please take a few minutes to complete a follow-up survey? Thank you!

Patient: ok.

System: our follow-up questions mainly include three aspects: ward environment, propaganda, and nursing. All of the following questions are ranked from 0 to 5, with 5 being the most satisfactory and 1 being the worst. We will record your questions at the end of the follow-up, and would you please help us complete the complete survey?

Patient: yes, no problem.

System: what is your score for the quiet and tidy environment of the ward? The full score is 5 points.

Patient: well, give it a five.

System: ok, may I ask how satisfied you are with the ward environment introduction, health guidance, and the matters needing attention after discharge, etc., given by the nurses during your stay in the hospital? Would you please rate

Patient: well, give it a five.

System: ok, please rate the nurses' responsibility, fit degree, injection technique, regular patrol, response speed, and other nursing work.

Patient: well, five points.

System: ok, what are your complaints about this hospitalization?

Patient: perhaps the most unsatisfactory part is that the doctor is too busy and has too few opportunities to communicate with patients.

System: do you have any other questions for feedback or consultation?

Patient: no.

System: thank you very much for your cooperation. We have recorded your problems and will reply to you as soon as possible. Please keep your mobile phone free and remember to review it on time.

**Table 1:** Automatically generated table for the dialogue in Textbox 1.

Parameter	Information
Basic information	Patient name, ID number, gender, age, discharge date, diagnosis, and caller location^a^
Dialogue result	System hang up after dialogue
Number of dials	1
Call duration (s)	97
Question 1 score	5
Question 2 score	5
Question 3 score	5
Total score	15
Feedback question	Doctors are too busy to communicate with patients
Follow-up date	2019/6/24

^a^Basic information is automatically extracted from the hospital information management system.

### Manual Follow-Up

Follow-up with the control group was performed by phone. Calls were made one by one depending on the availability of human resources. The communication contents were consistent with the AI-assisted follow-up group. Reports were recorded and uploaded manually. Feedback from patients were recorded by the operator and reported to surgeons and nurses.

### Evaluation Indicators

Evaluation indicators of follow-up included telephone connection rate, follow-up rate, feedback collection rate, and session duration. Telephone connection rate = (number of effective follow-ups + number of invalid follow-ups) / number of patients called × 100%. Follow-up rate = number of effective follow-ups / (number of effective follow-ups + number of invalid follow-ups) × 100%. Feedback collection rate = number of patients with effective feedback / number of effective follow-ups × 100%. Effective follow-up was defined as the complete collection of data in Table 1 (excluding the feedback question parameter), while invalid follow-up was defined as the absence or incomplete collection of data in Table 1. Effective feedback included patient feedback about nursing, health education, hospital environment, and medical consulting. Moreover, feedback content should be specific and constructive such as dissatisfaction with hospital food or postoperative wound rupture.

### Statistical Methods

Data analysis was performed using SPSS statistical software (version 23.0, IBM Inc). Pearson chi-square test or Fisher exact test was used to compare categorical variables (age, gender, medical condition, telephone connection rate, follow-up rate, and feedback rate). The normality of continuous variables (session duration) was tested with a modified Kolmogorov-Smirnov test. Unpaired *t* tests were performed for those following a normal distribution. All tests were two-sided. Data were considered to be statistically significant for *P*＜.05.

## Results

### Patient Characteristics

Patient characteristics for the two groups are shown in Table 2. Group differences in age, gender, and disease category were not statistically significant (Table 2).

**Table 2:** Comparison of patient characteristics in manual and artificial intelligence–assisted follow-up groups.

Characteristics		Manual follow-up group, n (%)		AI-assisted^a^ follow-up group, n (%)		Chi-square (*df*)		*P* value	
Number of patients		2656 (100)		270 (100)					
**Age (years)**						0.8 (3)		.86	
	<50		805 (30.3)		77 (28.5)				
	50-59		480 (18.1)		47 (17.4)				
	60-69		861 (32.4)		94 (34.8)				
	≥70		510 (19.2)		52 (19.3)				
**Gender**						1.7 (1)		.19	
	Male		994 (37.4)		112 (41.5)				
	Female		1662 (62.6)		158 (58.5)				
**Disease category**						4.1 (2)		.13	
	Spinal disease		1676 (63.1)		154 (57.0)				
	Joint disease		938 (35.3)		110 (40.7)				
	Other		42 (1.6)		6 (2.2)				

^a^AI-assisted: artificial intelligence–assisted.

### Cost-Effectiveness

As shown in Table 3, there was no significant difference in either telephone connection rate (manual: 2478/2656, 93.3%; AI-assisted: 249/270, 92.2%; *P*=.50) or follow-up rate (manual: 2301/2478, 92.9%; AI-assisted: 231/249, 92.8%; *P*=.96) between two groups. However, the feedback collection rate in the AI-assisted follow-up group was significantly higher than that in the manual follow-up group (manual: 68/2656, 2.5%; AI-assisted: 28/270, 10.3%; *P*<.001). The approximate session duration of the manual follow-up group varied from 60 seconds to 180 seconds, according to interviews with the operators. An extra 120 to 180 seconds were also necessary for material recording and uploading. Therefore, the average time spent on each patient in the manual follow-up group was approximately 3-6 minutes. We recorded the total time that the operators spent on randomly following 100 patients (9.3 hours). The average session duration of the AI-assisted follow-up group was 87.7 (SD 39.5) seconds. However, none of this time required human resources, and the AI system generated data reports automatically. In this way, the time spent on AI-assisted follow-up was close to 0 hours. Compared with manual follow-up, the AI-assisted follow-up were of shorter duration.

**Table 3:** Comparison of manual and artificial intelligence–assisted follow-up indicators.

Indicators		Manual follow-up		AI-assisted^a^ follow-up		Chi-square (*df*)		*P* value	
**Telephone connection**						0.4 (1)		.50	
	Number of effective follow-ups + invalid follow-ups		2478		249				
	Number of patients called		2656		270				
	Rate, %		93.3		92.2				
**Follow-up**						0.003 (1)		.96	
	Number of effective follow-ups + invalid follow-ups		2301		231				
	Number of effective follow-ups + invalid follow-ups		2478		249				
	Rate, %		92.9		92.8				
**Feedback collection**						47.1 (1)		＜.001	
	Number of patients with effective feedback		68		28				
	Number of effective follow-ups + invalid follow-ups		2656		270				
	Rate, %		2.5		10.3				
Time spent, hours per 100 patients		9.3		0		N/A^b^		N/A	

^a^AI-assisted: artificial intelligence–assisted.

^b^N/A: not applicable.

### Feedback Composition

**Figure 2:**
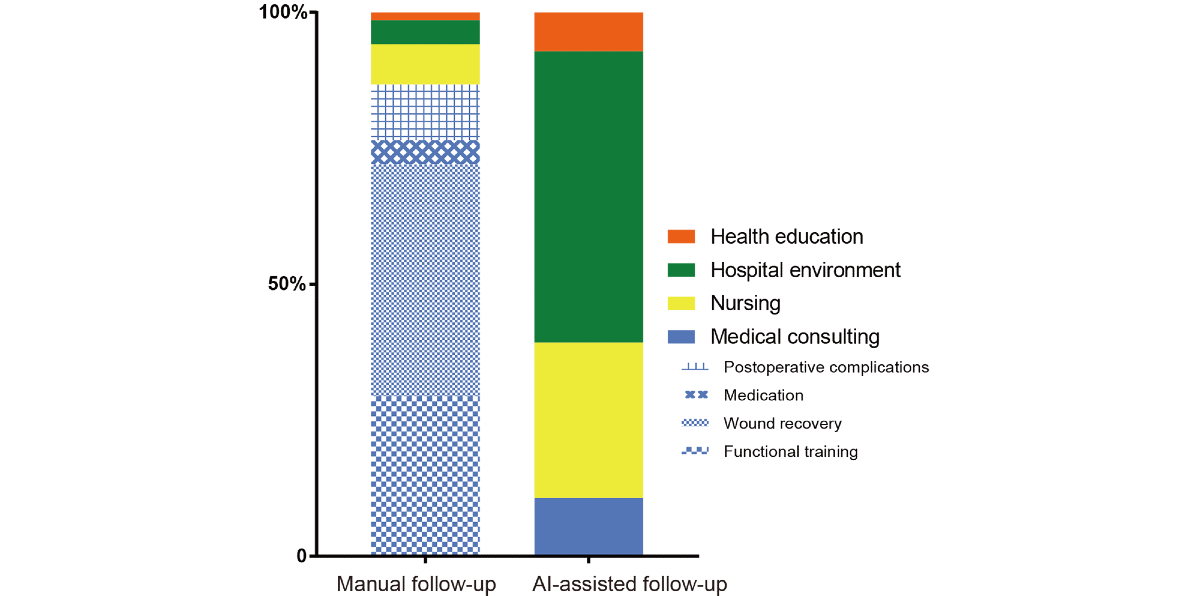
Composition of feedbacks in AI-assisted follow-up group and manual follow-up group.

The composition of feedback content is shown in Figure 2. In the manual follow-up group, 87.0% of the feedback were medical consultation–related, including functional training, wound recovery, medication usage and postoperative complication (29.5%, 42.7%, 4.5%, and 10.3%, respectively). Feedback related to nursing, health education and hospital environment accounted 7.3%, 1.4%, and 4.3%, respectively. In the AI-assisted follow-up group, only 10.7% of the feedback were related to medical consultation and most of the feedback focused on hospital environment, nursing, and health education (53.6%, 28.6%, and 7.1%, respectively).

## Discussion

### Principal Findings

In this paper, we put forward a new methodology for postoperative data collection for follow-up of patients who had undergone orthopedic surgery. The AI-assisted follow-up system is intended to facilitate follow-up efficiency via machine learning, speech recognition, and human voice simulation technology.

In the cost-effectiveness analysis, our study found that there was no significant difference in the telephone connection rate and follow-up rate between the two groups, suggesting the effectiveness of AI was not inferior to the traditional manual method. AI-assisted follow-up could replace traditional manual follow-up to some extent. At PUMCH, follow-up was traditionally completed by nurses or telephone operators where the average time spent on each patient was about 3-6 minutes. The AI-assisted system, however, was able to follow up 5-7 patients simultaneously using telephone relay technology. Thousands of patients could be followed up daily while the time spent on AI-assisted follow-up was close to 0 hours, saving a lot of manpower and human resources.

AI-assisted follow-up did improve the feedback rate, but the composition of feedback was different between the two groups. Feedback from the AI-assisted follow-up group were mainly related to nursing, health education, and hospital environment; only 11% was related to medical consulting. Conversely, this number was 87% in the manual follow-up group. After interviewing the operators, we found a possible explanation to be the telephone operator (usually nurses) were more likely to record feedback that was difficult for them to respond directly, such as medical consultation. In order to improve follow-up efficiency, they tended to respond directly to feedback related to nursing, hospital environment, and health education. This feedback would not have been recorded in the follow-up materials. It also explains why the feedback rate was lower in the manual follow-up group. Another possible explanation was that compared with AI-assisted follow-up, patients could communicate more naturally and deeply with operators. As a result, they may have been more willing to put forward professional medical consultation feedback to operators. In the manual follow-up group, medical consulting could be divided into four categories while in the AI-assisted follow-up group, medical consultation was difficult classify because it was not pertinent.

### Limitations

Our work has several limitations. First, apart from phone calls, there are other communication methods that could be combined with AI, such as text messages, computer software, and smartphone apps. Anthony et al [15] invented an automated mobile phone messaging platform for orthopedic trauma patients that improved the responding rate after trauma procedures; however, many elderly patients were not familiar with texting. Therefore, texting might not be suitable for follow-up. Another option is the chatbot. It is a computer program or smartphone app based on AI that can communicate with people via auditory or text [15]. Medical chatbots have been used in disease diagnosis [16], management [17], and monitoring [18]. Recent research showed that chatbots were a convenient method to help patients address common concerns after ureteroscopy [19]. Integrating chatbots into the telemedicine system could be used to help assess disease conditions and provide self-care recommendations for patients [20]. Therefore, future work may include developing a chatbot software or app for additional medical purposes and to provide better personal service.

The second limitation of this study is that the probation period of the AI-assisted follow-up system is not too long. It is generally believed that with the increasing of machine learning time, the AI-assisted system would become more intelligent. Therefore, we should pay more attention to this system in the future.

### Conclusions

In this research, we found that the effectiveness of AI-assisted follow-up was not inferior to the manual follow-up. Moreover, human resources costs could be saved with the assistance of artificial intelligence. Compared with manual follow-up, AI-assisted follow-up obtains more comprehensive feedback, but feedback lacks depth and pertinence. Therefore, the application of an AI-assisted follow-up system in hospital ward management has the potential to improve telemedicine follow-up service and patient satisfaction.
